# The Dual Role of Scavenger Receptor Class A in Development of Diabetes in Autoimmune NOD Mice

**DOI:** 10.1371/journal.pone.0109531

**Published:** 2014-10-24

**Authors:** Mami Shimizu, Hisafumi Yasuda, Kenta Hara, Kazuma Takahashi, Masao Nagata, Koichi Yokono

**Affiliations:** 1 Department of General Internal Medicine, Kobe University Graduate School of Medicine, Chuo-ku, Kobe, Japan; 2 Division of Health Sciences, Department of Community Health Sciences, Kobe University Graduate School of Health Sciences, Suma-ku, Kobe, Japan; 3 Division of Diabetes and Metabolism, Department of Internal Medicine, Iwate Medical University School of Medicine, Morioka, Japan; 4 Division of Internal Medicine and Diabetes, Kakogawa West City Hospital, Kakogawa, Japan; Dompé, United States of America

## Abstract

Human type 1 diabetes is an autoimmune disease that results from the autoreactive destruction of pancreatic β cells by T cells. Antigen presenting cells including dendritic cells and macrophages are required to activate and suppress antigen-specific T cells. It has been suggested that antigen uptake from live cells by dendritic cells via scavenger receptor class A (SR-A) may be important. However, the role of SR-A in autoimmune disease is unknown. In this study, SR-A^−/−^ nonobese diabetic (NOD) mice showed significant attenuation of insulitis, lower levels of insulin autoantibodies, and suppression of diabetes development compared with NOD mice. We also found that diabetes progression in SR-A^−/−^ NOD mice treated with low-dose polyinosinic-polycytidylic acid (poly(I∶C)) was significantly accelerated compared with that in disease-resistant NOD mice treated with low-dose poly(I∶C). In addition, injection of high-dose poly(I∶C) to mimic an acute RNA virus infection significantly accelerated diabetes development in young SR-A^−/−^ NOD mice compared with untreated SR-A^−/−^ NOD mice. Pathogenic cells including CD4^+^CD25^+^ activated T cells were increased more in SR-A^−/−^ NOD mice treated with poly(I∶C) than in untreated SR-A^−/−^ NOD mice. These results suggested that viral infection might accelerate diabetes development even in diabetes-resistant subjects. In conclusion, our studies demonstrated that diabetes progression was suppressed in SR-A^−/−^ NOD mice and that acceleration of diabetes development could be induced in young mice by poly(I∶C) treatment even in SR-A^−/−^ NOD mice. These results suggest that SR-A on antigen presenting cells such as dendritic cells may play an unfavorable role in the steady state and a protective role in a mild infection. Our findings imply that SR-A may be an important target for improving therapeutic strategies for type 1 diabetes.

## Introduction

Human type 1 diabetes (T1D) is an autoimmune disease that results from the autoreactive destruction of pancreatic β cells by T cells and the subsequent loss of insulin production [1]. It is thought that β-cell antigens are taken up through surface receptors on antigen-presenting cells (APCs). APCs such as dendritic cells (DCs) and macrophages are required to activate and suppress antigen-specific T cells. Nonobese diabetic (NOD) mice serve as a spontaneous model system for studying the mechanisms involved in the initiation and propagation of the autoimmune response of human T1D [2]. In NOD mice, pancreatic β cells are destroyed by chronic autoimmune response mainly mediated by autoreactive CD4^+^ T cells and CD8^+^ T cells. The effector T cells are β cell-reactive CD4^+^ T cells producing Th1 cytokines such as IFN-γ and IFN-γ-producing cytotoxic CD8^+^ T cells. The cytotoxicity of β cells depends on the effects of effector T cells via FasL/Fas, perforin/granzyme B, or NO and cytokines. In contrast, CD4^+^ Foxp3^+^ T cells in CD4^+^ CD25^+^ T cell population are considered to be regulatory T cells (Treg), which play a crucial role in protecting β cells from autoimmune destruction. However, in NOD mice, the balance between effector T cells and Treg shifts to effector T cells, and finally leads to disease onset [2], [3]. A panel of studies on prevention and reversal of T1D in NOD mice have been reported so far [3]–[6]. In particular, reversal of T1D is clinically more important, but the studies on reversal in mouse models are not successfully applied in humans yet.

Scavenger receptors (SRs) are classified into eight classes (A–H) by differences in their structures. Scavenger receptor class A (SR-A) is present on DCs and macrophages. It has been suggested that antigen uptake from live cells by DCs via SR-A may be important [7]–[9]. SR-A is implicated in atherogenesis as a result of receptor-mediated uptake of modified low-density lipoproteins. SR-A^−/−^ mice are reported to show increased susceptibility to infection with *Listeria monocytogenes* and herpes simplex virus type 1 [10].

Toll-like receptors (TLRs) have been reported to be expressed on DCs and macrophages and are considered to be fundamental sensors for innate immunity. They recognize pathogens such as bacteria, viruses, fungi and endogenous DNA or RNA. They also have been reported to control adaptive immunity. TLR3 located in cellular endosomes detects viral nucleic acids and is activated through uptake of extracellular virus-derived RNA molecules. Polyinosinic–polycytidylic acid (poly(I∶C)) is a double-stranded RNA (dsRNA) analogue and is considered to be a TLR3 ligand [11].

Recently, it was reported that SR-A is a cell surface receptor for dsRNA and that extracellular dsRNA is recognized and internalized by SR-A [12]–[14]. It was also reported that while diabetes development was completely prevented in MyD88^−/−^ NOD mice, the deletion of TLR3, which is not associated with MyD88, could not suppress diabetes development in NOD mice [15], [16].

To investigate whether SR-A plays a crucial role in the transport of dsRNA to TLR3, we studied diabetes progression in NOD and SR-A^−/−^ NOD mice in the presence or absence of poly(I∶C) treatment.

## Materials and Methods

### Ethics

This study was approved by the Institutional Animal Care and Use Committee of Kobe University School of Medicine and carried out according to the Kobe University Animal Experimentation Regulation (P131001). All efforts were made to minimize suffering.

### Mice

**Figure 1:**
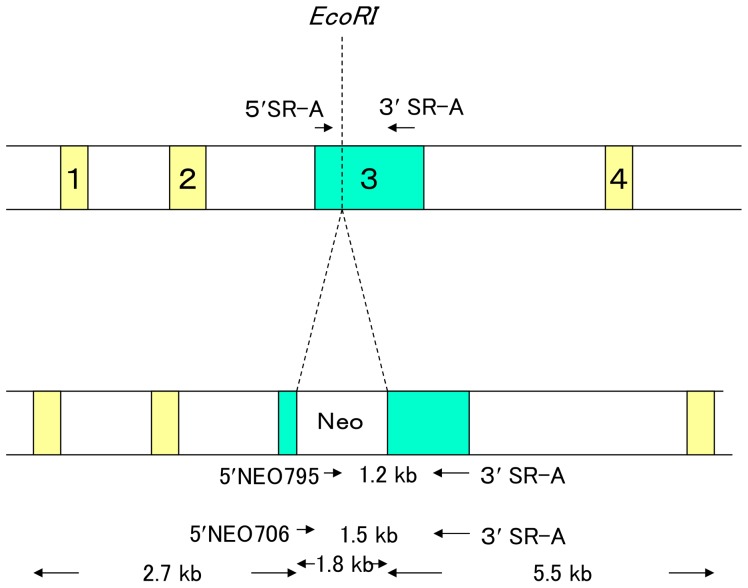
Targeting of the mouse SR-A gene. The SR-A genomic fragment was composed of exons 3 to 5. A 1.8-kb *Xho*I–*Sac*I fragment of pGK1 neo-poly(A)^+^ was inserted into the *Eco*RI site in exon 3.

The original SR-A knockout (SR-A^−/−^) mice were produced by Kodama and colleagues as described previously [10], using 129Sv ES cells microinjected into C57BL/6J blastocysts and embryos transferred into the uteri of ICR mice (**Figure 1**). These original SR-A^−/−^ mice with backgrounds from 129, C57BL/6, and ICR mice were backcrossed 10 generations (BC10) with NOD/Shi/Kbe mice in the Institute for Experimental Animals, Kobe University School of Medicine, and Tohoku University School of Medicine to generate the SR-A^−/−^ NOD mice. Experimental animals were produced by brother–sister mating of heterozygous mice at BC10 to obtain homozygous knockout mice. All animals were housed in specific pathogen-free facilities and handled under the Guidelines for Animal Experimentation of Kobe University School of Medicine.

### Antibodies and reagents

Fluorescein isothiocyanate (FITC)-conjugated anti-mouse CD25 monoclonal antibody (7D4), the peridinin chlorophyll protein complex (PerCP)-conjugated anti-mouse CD4 monoclonal antibody (L3T4), phycoerythrin (PE)-conjugated anti-mouse CD11c monoclonal antibody (HL3) and PE-conjugated anti-mouse CD4 monoclonal antibody (L3T4) were purchased from BD Biosciences Pharmingen (San Diego, CA). PE-conjugated anti-mouse Foxp3 monoclonal antibody (FJK-16s) was purchased from eBioscience (San Diego, CA). FITC-conjugated anti-mouse CD204 (SR-A) monoclonal antibody (2F8) was purchased from AbD Serotec (Oxford, UK). Anti-CD11c (N418) and anti-CD11b microbeads were purchased from Miltenyi Biotec (Auburn, CA). Anti-CD3εAb was purchased from R&D Systems (Minneapolis, MN).

### Lipid profiles

Sera was obtained from SR-A^−/−^ NOD mice and NOD mice at 12–17 weeks of age by retro-orbital puncture after a 18 hrs fast. Serum levels of total cholesterol, LDL-C and triglyceride were measured by SRL laboratory (Osaka, Japan).

### Genotype analysis

Genomic DNA was extracted from mouse tails. Genotyping for the SR-A^−/−^ gene and wild-type gene were performed using PCR amplification. In addition, mice were genotyped by PCR with microsatellite markers to fix the NOD diabetogenic idd 1–19 loci and the non-NOD genomic regions flanking the SR-A gene knockout. The PCR products were electrophoresed on agarose gels and visualized by ethidium bromide staining.

### Assessment of diabetes development

Blood glucose levels were measured weekly with the Glutest Ace (Sanwakagaku, Nagoya, Japan) blood glucose monitoring system. Glucose levels >250 mg/dl for 2 consecutive days were defined as diabetes.

### Histology

The pancreas was harvested from five 15–20-week-old prediabetic mice, fixed in 10% formalin, and embedded in paraffin. Five-micrometer-thick sections were cut, stained with hematoxylin and eosin, and examined by light microscopy. The degree of insulitis was scored from 0 to 4 with 0 (within normal limits, absent), 1 (0–25%, islets showing lymphocyte infiltration), 2 (25%–50%), 3 (50%–75%), and 4 (75%–100%) by a reader blinded to the category of the mice.

### Detection of IAA

IAA was estimated in the serum of the prediabetic mice at 8, 12, 16, and 20 weeks of age as previously described [17]. ^125^I-insulin (GE Healthcare, Little Chalfont, UK) was incubated with 6 µl of serum with and without old human insulin, and Protein A/G Sepharose was added to the incubation in a 96-well plate format for each serum. Finally, 50 µl of scintillation liquid was added to each well and radioactivity was counted with a 96-well plate scintillation β-counter (PerkinElmer; Waltham, MA). The result was expressed as an index: index = (sample Δ cpm – negative control Δ cpm)/(positive Δ cpm – negative control Δ cpm). A value of 0.01 or greater was considered positive.

### Isolation of macrophages from SR-A^−/−^ NOD mice

SR-A^−/−^ NOD mice were sacrificed at 8 weeks of age. CD11b^+^ cells isolated from the abdominal cavity of mice were sorted with an autoMACS magnetic cell sorter (Miltenyi Biotec, GmbH, Bergisch-Gladbach, Germany).

### Generation of bone marrow-derived dendritic cells (BMDCs)

DCs were generated from mouse bone marrow as described previously [18], [19]. Briefly, bone marrow cells were isolated from 7–9-week-old SR-A^−/−^ NOD mice or NOD mice. CD11c^+^ cells were isolated from cultured cells with an autoMACS magnetic cell sorter and were designated as BMDCs.

### Flow cytometric analysis

The fluorescence intensity of the stained cells was analyzed on a FACS 440 flow cytometer (Becton Dickinson, San Jose, CA).

### RNA isolation and cDNA synthesis

mRNA was extracted from spleen cells and BMDCs with a Micro-FastTrack mRNA isolation kit (Invitrogen, NV, Leek, The Netherlands) and mRNA was reverse-transcribed with a cDNA cycle kit(Invitrogen), while using oligo-dT primers and AMV reverse transcriptase to generate cDNA for use as a template in PCR amplifications.

### PCR analysis

The PCR analysis was carried out using cDNA samples and genomic DNA for analysis of SR-A, 20 µM of each primer, and 1.25 U of Ex*Taq* polymerase (Takara Shuzo, Shiga, Japan) in a 50-µl final volume. Samples were amplified with an initial 4-min denaturation at 95°C, followed by 35 cycles of 30 sec at 95°C, 30 sec at 65°C, and 1 min at 72°C, with 10 min at 72°C on the last cycle in a Gene Amp PCR System 9700 (Perkin-Elmer/Cetus Corp, Norwalk, CT). The upstream and downstream primers were for 3′SR-A: TCAGGTGCAGAACACTTCAGT and for 5′NEO706: TGCTTTGCTGTAGATTCACGG or 5′NEO795: GCTGTCCATCTGCACGAGAC, respectively. The PCR products were electrophoresed on agarose gels and visualized by ethidium bromide staining.

### In vivo treatment

Polyinosinic–polycytidylic acid (Poly(I∶C)), a ligand for TLR3, was purchased from Sigma-Aldrich (St. Louis, MO). Poly(I∶C) was given intraperitoneally for 28 days (starting at 3–4 weeks of age) at 100 µg/mouse or 300 µg/mouse.

### In vitro cytokine production from BMDCs with or without poly(I∶C)

Purified BMDCs (1×10^6^/500 µl, 1 ml) from the NOD mice or SR-A^−/−^ NOD mice were incubated with poly(I∶C) (10, 50 µg/ml) or medium for 5 h and 10 h. TNF-α cytokine was measured from the 10 h culture supernatant samples and IFN-β cytokine was measured from the 5 h culture supernatant samples. Levels of TNF-α and IFN-β were measured using commercially available enzyme-linked immunosorbent assay (ELISA) kits from R&D Systems.

### Phenotype of splenocytes in NOD and SR-A^−/−^NOD mice treated with or without poly(I∶C)

Poly(I∶C) was given intraperitoneally for 14 days (starting at 4 weeks of age) at 100 µg/mouse or 300 µg/mouse. Splenocytes were isolated from mice and costained with PE-conjugated anti-CD4 and FITC-conjugated anti-CD25. The percentage of CD4^+^CD25^+^ cells was analyzed by flow cytometry. Splenocytes were also costained with PerCP-conjugated anti-CD4 and PE-conjugated anti-Foxp3 after permeabilizing according to the manufacturer's instructions. The percentage of CD4^+^Foxp3^+^ cells was analyzed by flow cytometry.

### In vitro stimulation of splenocytes from NOD and SR-A^−/−^ NOD mice with or without poly(I∶C) treatment

Purified splenocytes (1×10^6^/200 µ1) from the NOD mice or SR-A^−/−^ NOD mice treated or not treated with poly(I∶C) were resuspended in culture medium and transferred to each well of a round-bottom 96-well plate. Then, anti-CD3εAb was added to each well (final concentration 2.5 µg/ml). No stimulant was added to control wells. Then the cells were cultured for 72 h at 37°C in a humidified 5% CO_2_ atmosphere. The supernatant was collected at the end of culture and frozen at −30°C until cytokine assay. The concentration of IL-17 and IFN-γ were evaluated by ELISA as recommended by the assay manufacturer (R&D Systems).

### Statistical analysis

Survival curves were analyzed with a log-rank test using the Kaplan–Meier method. Mann-Whitney *U* tests were used to compare the mean values of groups. Graph Pad Prism 4 for Windows (GraphPad Software, San Diego, CA) was used for statistical analysis.

## Results

### SR-A expression in SR-A^−/−^ NOD mice and littermates

**Figure 2:**
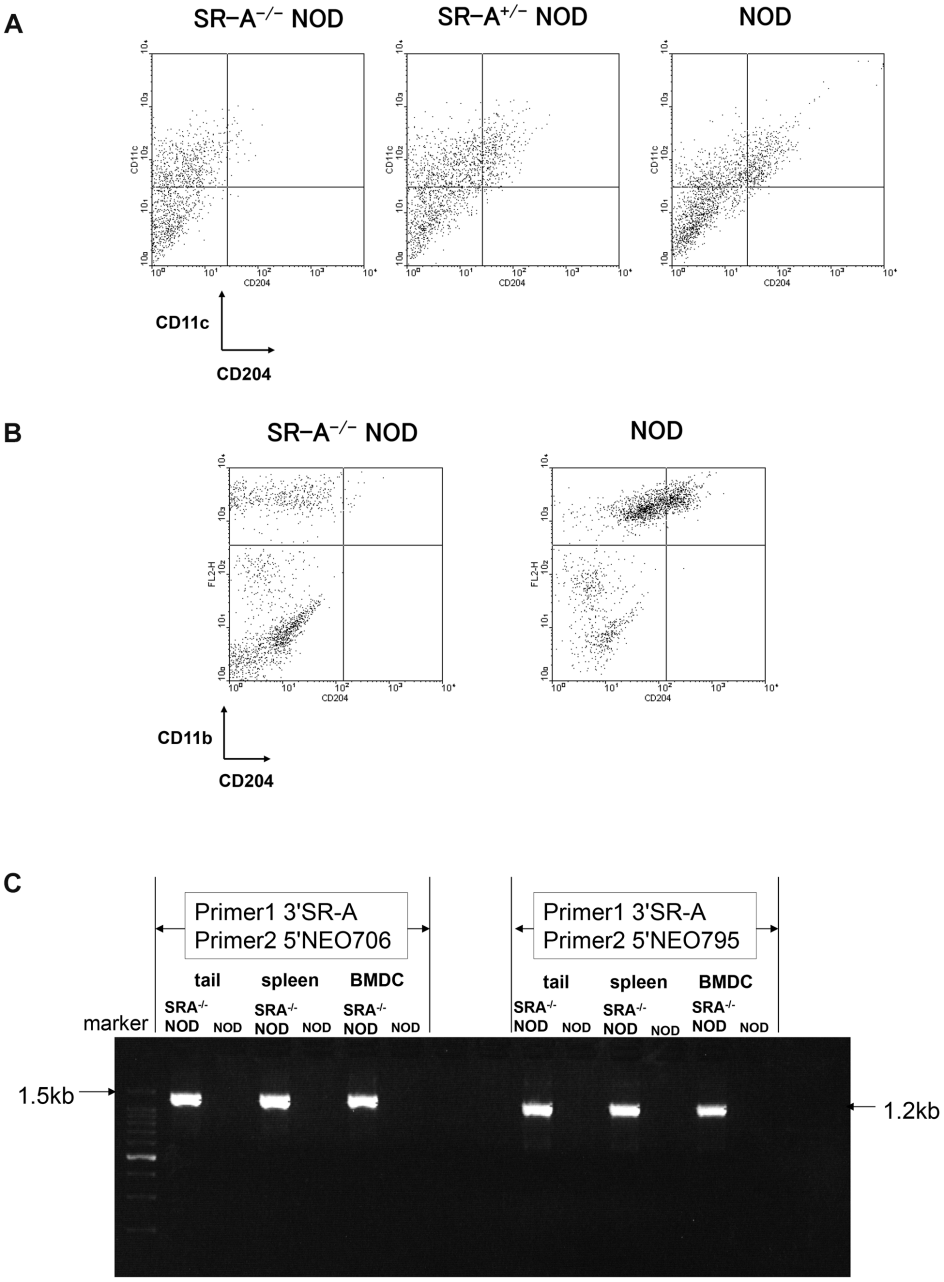
Disruption of SR-A confirmed by flow cytometry and by PCR analysis in SR-A^−/−^ NOD mice. DCs (A) and macrophages (B) were analyzed by flow cytometry. Bone marrow cells isolated from 7–9-week-old SR-A^−/−^ NOD mice or NOD mice were cultured with granulocyte-macrophage colony-stimulating factor and IL-4 for 7 days and DCs were isolated using magnetic beads. Macrophages were obtained from the abdominal cavity of 8-week-old mice. Representative data for the double staining of CD11c and CD204 (SR-A) or CD11b and CD204 are shown for SR-A^−/−^ NOD mice. (C) Neo gene–containing PCR products (1.5 kb and 1.2 kb) from genomic DNA of tails and mRNA from spleen cells and BMDCs were detected in SR-A^−/−^ NOD mice, but not in NOD mice. PCR analysis confirmed that SR-A gene was indeed deficient in SR-A^−/−^ NOD mice.

To explore the role of SR-A in the development of T1D, we generated SR-A^−/−^ NOD mice by crossing the original SR-A^−/−^ mice with the NOD strain for 10 generations using speed congenic methods. We examined the pattern of SR-A expression on macrophages and DCs of prediabetic animals by flow cytometry. SR-A molecules were absent from the surface of both CD11c positive DCs (**Figure 2A**) and CD11b positive macrophages (**Figure 2B**) from SR-A^−/−^ NOD mice. Neo gene–containing PCR products (1.5 kb and 1.2 kb) were detected in SR-A^−/−^ NOD mice, but not in NOD mice. PCR analysis confirmed that SR-A gene was indeed deficient in SR-A^−/−^ NOD mice (**Figure 2C**)

### Lipid profiles

The levels of total cholesterol, LDL-C, triglyceride were measured in SR-A^−/−^ NOD mice and NOD mice, respectively. The results indicate that serum levels of total cholesterol and LDL-C in SR-A^−/−^ NOD mice are relatively higher than those in NOD mice, but not significantly (*p*>0.05 by Mann–Whitney *U* test) (**Table 1**). We found that there were no significant differences in metabolic condition between SR-A^−/−^ NOD mice and NOD mice.

**Table 1:** Lipid profiles.

	NOD mouse		SR-A^−/−^ NOD mouse		
	Mean	SD	Mean	SD	p value
LDL-C (mg/dL) 12–17 w (n = 6)	9.667	1.633	10.5	1.378	0.3691
Total cholesterol (mg/dL) 12–17 w (n = 10)	91.1	8.386	95.3	14.83	0.9698
Triglyceride (mg/dL) 12–17 w (n = 10)	27.6	10.69	26.5	10.7	0.7908

(p value by Mann–Whitney U test).

### Protection against development of diabetes in female SR-A^−/−^ NOD mice

**Figure 3:**
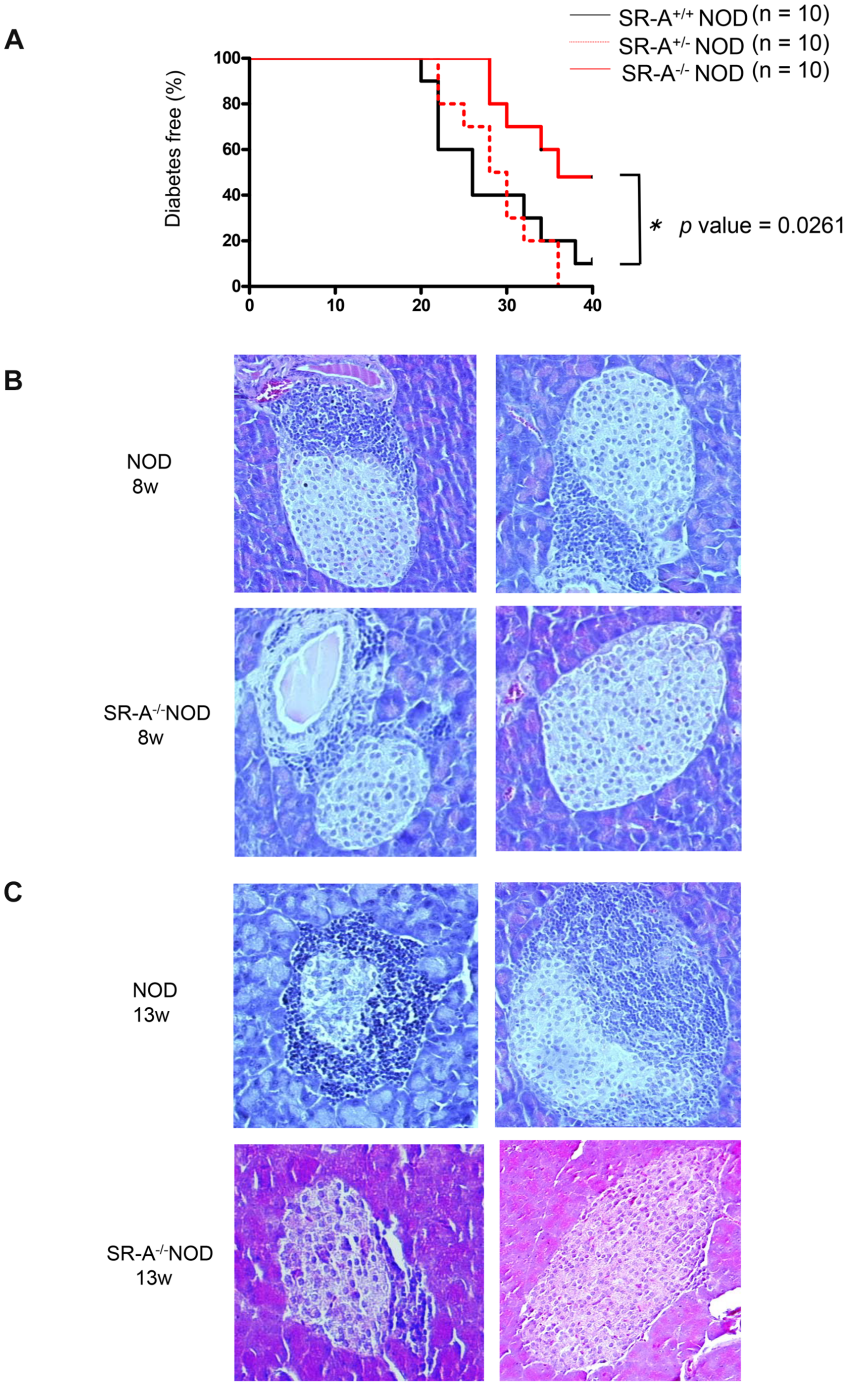
Protection against development of diabetes in female SR-A^−/−^ NOD mice. (A)Blood glucose was monitored weekly in each of 10 mice starting when mice reached 4 weeks old and continuing up to 40 weeks of age. Glucose levels >250 mg/dl for 2 consecutive days were defined as diabetes. The development of diabetes was significantly suppressed in female SR-A^−/−^ NOD mice (* *p* value = 0.0261, compared with littermates, log-rank test). Representative histological appearances are shown for SR-A^−/−^ NOD mice and NOD mice, (B) at 8 weeks of age, and (C) at 13 weeks of age.

To examine the development of diabetes in SR-A^−/−^ NOD mice, glucose levels were monitored weekly in the female SR-A^−/−^ NOD mice and SR-A^+/−^ NOD mice compared with their wild type littermates. As shown in **Figure 3A**, the onset of diabetes in SR-A^−/−^ NOD mice was significantly suppressed compared with the wild type littermates (* *p* value = 0.0261 by log-rank test). The cumulative diabetes incidence in SR-A^−/−^ NOD mice was 50% at 40 weeks of age, whereas that in SR-A^+/−^ NOD mice and NOD mice of the same age was 100% and 90%, respectively. In addition, we performed experiments in two other T1D models; cyclophosphamide (CY)-induced diabetes model (**Figure S1**) and multiple low-dose STZ (MLDS)-induced diabetes model (**Figure S2**). We found that the results were not the same as that in spontaneous SR-A^−/−^ NOD mice, suggesting that the differences among three models may depend on each different mechanism of T1D model.

### Insulitis development in SR-A^−/−^ NOD mice

**Figure 4:**
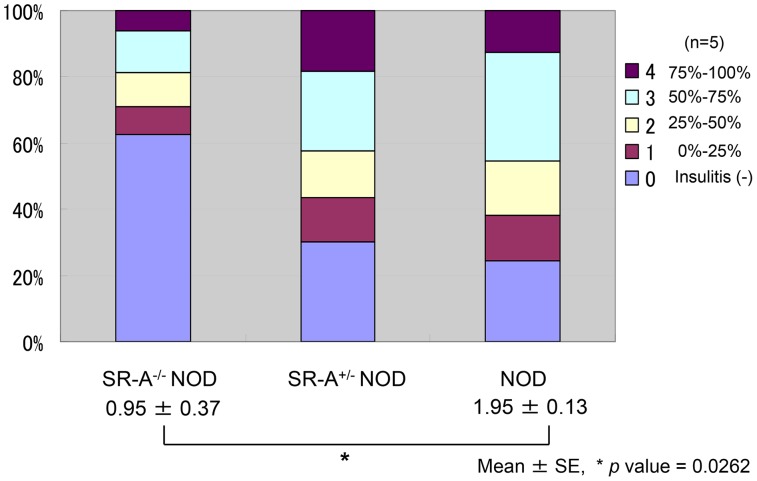
Suppression of insulitis in SR-A^−/−^ NOD mice. The severity of islet inflammation was quantified by the degree of insulitis. Islets were categorized as follows: 0 (within normal limits, absent), 1 (the percentage of islet lymphocyte infiltration, 0–25%), 2 (25%–50%), 3 (50%–75%), and 4 (75%–100%). Five mice (15–20 weeks of age, average age 17.5 weeks) were analyzed and an insulitis score was calculated. The score was significantly suppressed in SR-A^−/−^ NOD mice compared with littermates (* *p* value = 0.0262 by Mann–Whitney *U* test).

To assess whether resistance to the development of diabetes was accompanied by reduced pancreatic islet inflammation, pancreatic sections from 10–15-week-old SR-A^−/−^ NOD, SR-A^+/−^ NOD and NOD mice were examined. Histological examination showed typical mononuclear cell infiltration of the pancreatic islets. This infiltration was observed in a high percentage of the examined islets in the SR-A^+/−^ NOD and NOD mice, while the severity of insulitis was significantly suppressed in the SR-A^−/−^ NOD pancreas, with a substantial reduction in intra-islet infiltration (* *p* value = 0.0262 by Mann–Whitney *U* test) (**Figure 3B**, **Figure 3C**, **Figure 4**). In addition, C-peptide levels are higher in SR-A^+/−^ NOD mice than in NOD mice, suggesting that C-peptide level is negatively related with insulitis and diabetes incidence in each mouse (**Figure S3**). These results indicated that the suppression of diabetes development in SR-A^−/−^ NOD mice was accompanied by attenuation of insulitis.

### Production of insulin autoantibodies (IAA) in SR-A^−/−^ NOD mice

**Figure 5:**
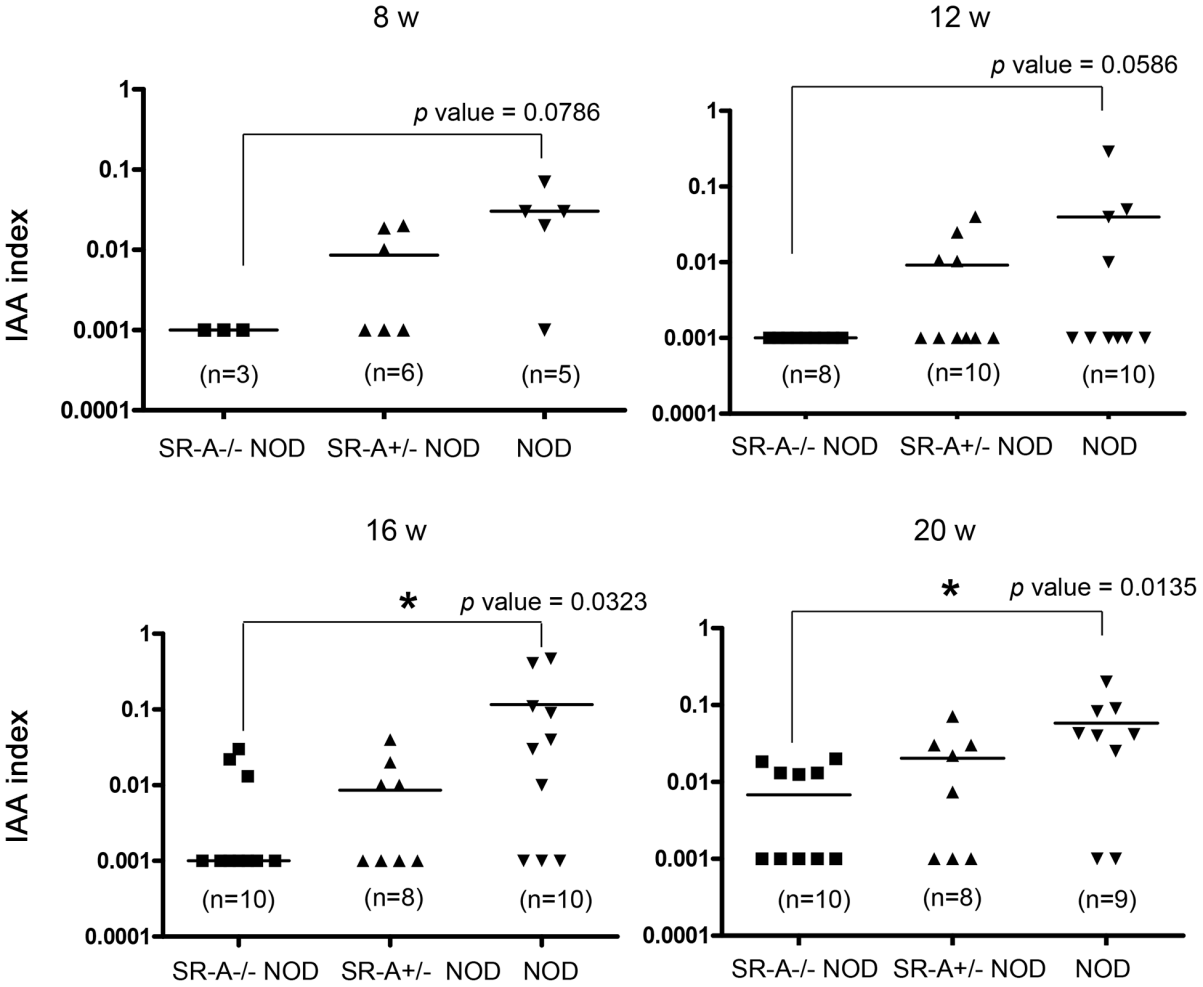
Diminished production of insulin autoantibodies (IAA) in SR-A^−/−^ NOD mice. Sera from 8-, 12-, 16-, and 20-week-old SR-A^−/−^ NOD, SR-A^+/−^ NOD and NOD female littermates were collected and analyzed by our standard IAA assay. Levels of IAA were significantly lower than NOD mice in 16- and 20-week-old SR-A^−/−^ NOD littermates (* *p*<0.05 by Mann–Whitney *U* test).

To evaluate the role of SR-A in the autoimmune process, we further measured the levels of IAA in sera from SR-A^−/−^ NOD, SR-A^+/−^ NOD and NOD mice by our standard radioassay. A significant reduction of IAA levels was observed in SR-A^−/−^ NOD mice compared with NOD mice at 16 and 20 weeks of age, correlating with the degree of insulitis (* *p*<0.05 by Mann–Whitney *U* test) (**Figure 5**). These results clearly suggested that SR-A deficiency affected the autoimmune process in NOD mice.

### Analysis of splenocyte phenotype and cytokine production

**Figure 6:**
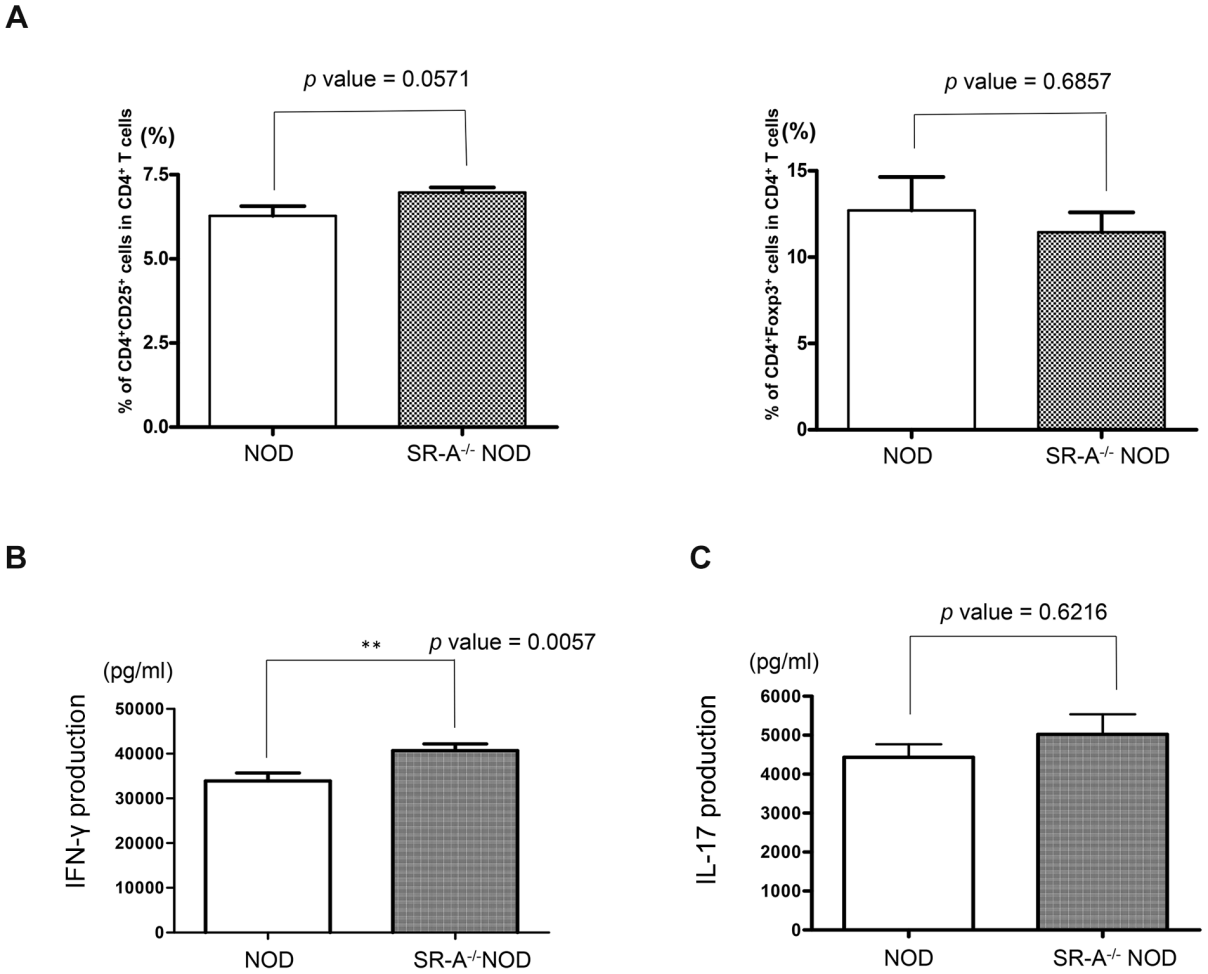
Phenotype and cytokine production of splenocytes from SR-A^−/−^ NOD mice. (A) Splenocytes from 6-week-old NOD or SR-A^−/−^ NOD mice were stained with antibodies against CD4, CD25 and Foxp3. A representative example of separate experiments is shown. Levels of CD4^+^CD25^+^ pathogenic cells and CD4^+^Foxp3^+^ regulatory cells in SR-A^−/−^ NOD mice were similar to those in NOD mice. (NOD mice; n = 4, SR-A^−/−^ NOD mice; n = 4), mean ± SD. CD4^+^CD25^+^ T cells; *p* value = 0.0571. CD4^+^Foxp3^+^ cells; *p* value = 0.6857. Cytokine profiles were examined after anti-CD3εAb stimulation of splenocytes from 6-week-old NOD and SR-A^−/−^ NOD mice without poly(I∶C) treatment. (B) IFN-γ production in SR-A^−/−^ NOD mice was higher than that in NOD mice. (NOD mice; n = 9, SR-A^−/−^ NOD mice; n = 10), mean ± SD. ** *p* value = 0.0057. (C) IL-17 production in SR-A^−/−^ NOD mice did not differ from that in NOD mice. (NOD mice; n = 5, SR-A^−/−^ NOD mice; n = 8), mean ± SD, *p* value = 0.6216.

Splenocytes from 6-week-old NOD and SR-A^−/−^ NOD mice were analyzed using flow cytometry to examine whether the spleen cells had a pathogenic or regulatory phenotype. The percentages of pathogenic CD4^+^CD25^+^ T cells and CD4^+^Foxp3^+^ regulatory T cells (Tregs) in SR-A^−/−^ NOD mice were similar to those in NOD mice (**Figure 6A**). We analyzed the cytokine profiles after anti-CD3ε antibody stimulation of spleen cells from NOD and SR-A^−/−^ NOD mice without poly(I∶C) treatment. Interferon (IFN)-γ production in SR-A^−/−^ NOD mice was higher than in NOD mice (** *p* value = 0.0057 by Mann–Whitney *U* test) (**Figure 6B**) Interleukin (IL)-17 production in SR-A^−/−^ NOD mice showed no marked increase compared with that in NOD mice (**Figure 6C**).

### Treatment with poly(I∶C) accelerates diabetes progression in young SR-A^−/−^ NOD mice

**Figure 7:**
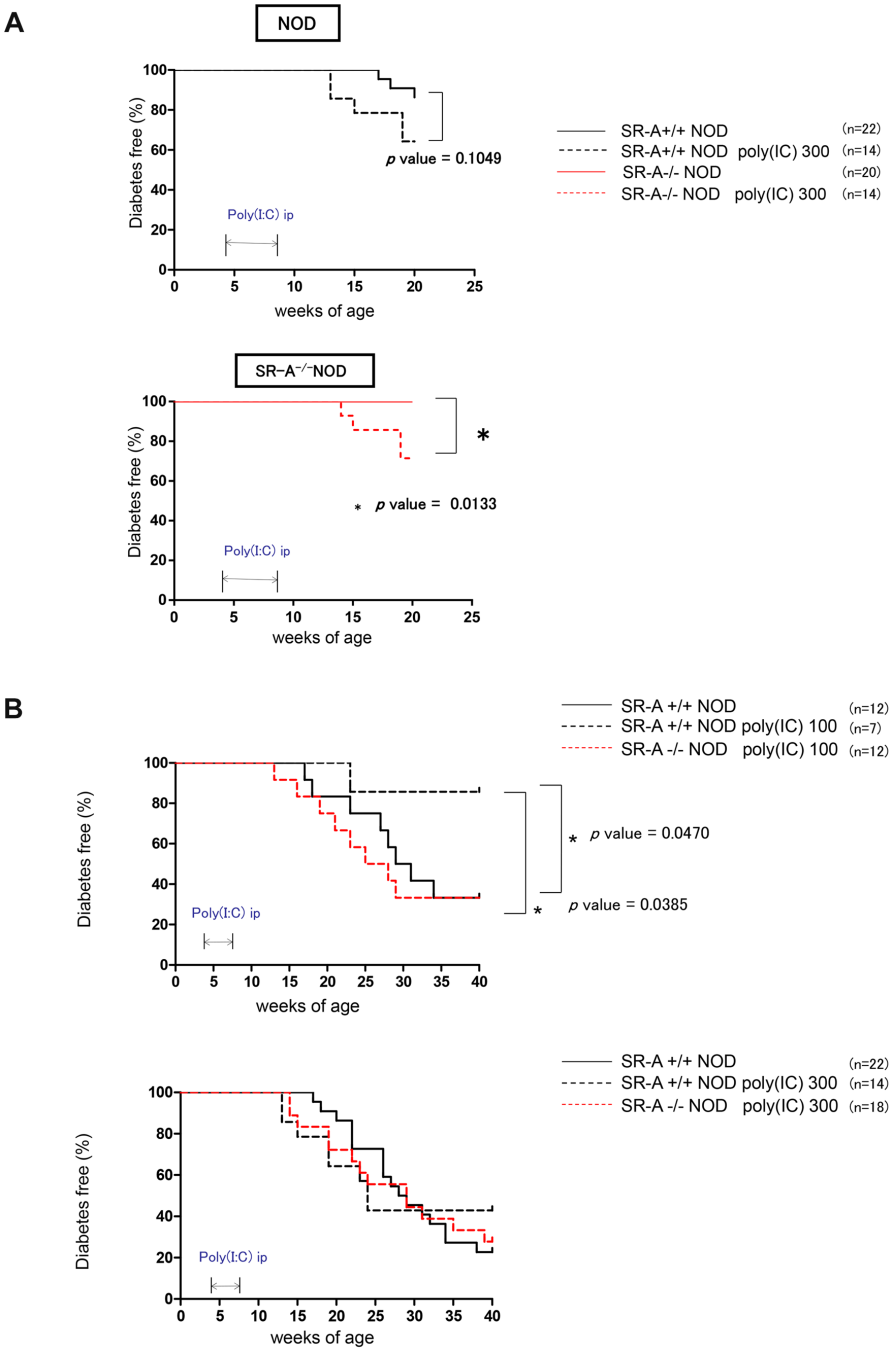
Incidence of diabetes in NOD or SR-A^−/−^ NOD mice treated with poly(I∶C). (A) We followed diabetes onset in SR-A^−/−^ NOD mice or NOD given high-dose poly(I∶C) (300 µg/mouse) for 28 days from 3–4 weeks of age at 20 weeks of age. Diabetes progression was significantly accelerated in SR-A^−/−^ NOD mice treated with poly(I∶C) compared with untreated SR-A^−/−^ NOD mice (* *p* value = 0.0133 by log-rank test). (B) We followed diabetes onset in SR-A^−/−^ NOD or NOD mice given low-dose poly(I∶C) (100 µg/mouse) or high-dose poly(I∶C) (300 µg/mouse) for 28 days from 3–4 weeks of age to 40 weeks of age. Diabetes progression was significantly suppressed by low-dose poly(I∶C) in NOD mice compared with untreated NOD mice (* *p* value = 0.0470 by log rank test). Diabetes progression in SR-A^−/−^ NOD mice treated with low-dose poly(I∶C) was significantly accelerated compared with that in NOD mice treated with low-dose poly(I∶C) (* *p* value = 0.0385 by log rank test). High-dose poly(I∶C) treatment could not suppress diabetes progression in SR-A^−/−^ NOD or NOD mice compared with untreated NOD mice. Diabetes progression was suppressed only by low-dose poly(I∶C) treatment in NOD mice.

SR-A^−/−^ NOD or NOD mice from 4 to 8 weeks of age were treated with poly(I∶C) and their blood glucose levels were monitored weekly (**Figure 7**). We followed diabetes development in SR-A^−/−^ NOD mice or NOD mice given high-dose poly(I∶C) (300 µg/mouse) for 28 days from 3–4 weeks of age. We found that 35.7% (5/14) of NOD mice treated with poly(I∶C) and 13.6% (3/22) of untreated NOD mice developed diabetes by 20 weeks of age. This showed no significant acceleration of diabetes development induced by high-dose poly(I∶C) in NOD mice up to 20 weeks of age. In contrast, 28.6% (4/14) of SR-A^−/−^ NOD mice treated with high-dose poly(I∶C) developed diabetes by 20 weeks of age compared with 0% of untreated SR-A^−/−^ NOD mice. Thus, diabetes progression was significantly accelerated in young SR-A^−/−^ NOD mice treated with poly(I∶C) compared with untreated SR-A^−/−^ NOD mice of the same age (* *p* value = 0.0133 by log-rank test) (**Figure 7A**). We subsequently examined diabetes development in SR-A^−/−^ NOD mice or NOD mice given low-dose poly(I∶C) (100 µg/mouse) for 28 days from 3–4 weeks of age. We found that 14.3% (1/7) of NOD mice treated with low-dose poly(I∶C) and 66.7% (8/12) of untreated NOD mice developed diabetes by 40 weeks of age. Diabetes progression was significantly suppressed by low-dose poly(I∶C) in NOD mice (* *p* value = 0.0470 by log-rank test). However, 66.7% (8/12) of SR-A^−/−^ NOD mice treated with low-dose poly(I∶C) developed diabetes by 40 weeks of age. This showed a marked acceleration of diabetes development caused by low-dose poly(I∶C) in SR-A^−/−^ NOD mice, with an incidence of diabetes similar to that of untreated NOD mice by 40 weeks of age. Diabetes progression in SR-A^−/−^ NOD mice treated with low-dose poly(I∶C) was significantly accelerated compared with that in NOD mice treated with low-dose poly(I∶C) (* *p* value = 0.0385 by log-rank test) (**Figure 7B**). We also followed diabetes development in SR-A^−/−^ NOD mice or NOD mice given high-dose poly(I∶C) 300 µg/mouse for 28 days from 3–4 weeks of age. We found that 72.2% (13/18) of SR-A^−/−^ NOD treated with high-dose poly(I∶C) developed diabetes by 40 weeks of age. This showed a marked acceleration of diabetes by high-dose poly(I∶C) in SR-A^−/−^ NOD mice, with an incidence similar to that of untreated NOD mice, 81.8% (18/22) of which developed diabetes by 40 weeks of age (**Figure 7B**). Similarly, 57.1% (8/14) of NOD mice treated with high-dose poly(I∶C) developed diabetes by 40 weeks of age. Cellular infiltration of mononuclear cells in islets were observed in both SR-A^−/−^ NOD mice and NOD mice treated with high-dose poly (I∶C) (**Figure S4**). Diabetes progression was suppressed only by low-dose poly(I∶C) treatment in NOD mice (**Figure 7B**).

### Cytokine production from poly(I∶C)-treated BMDCs in vitro

**Figure 8:**
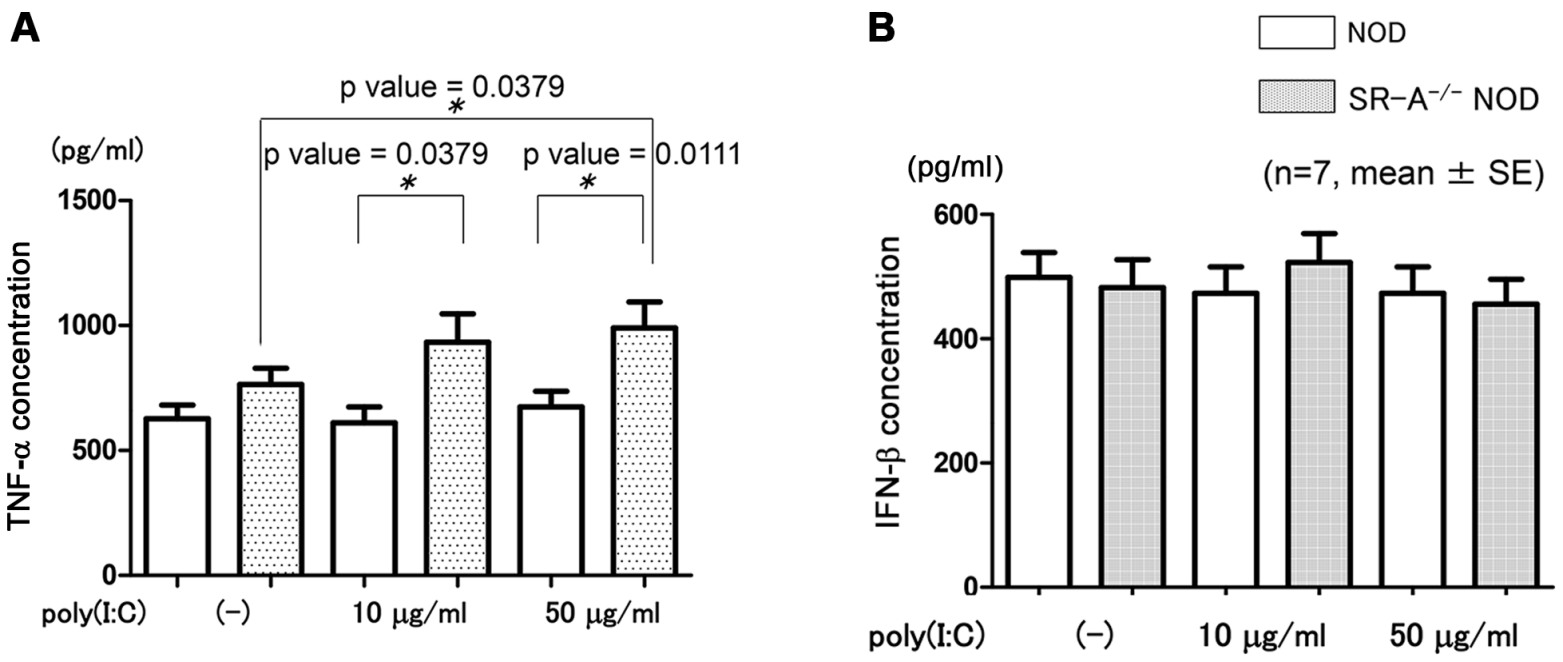
Type I IFN production from BMDCs stimulated with poly(I∶C). BMDCs from 7–9-week-old NOD and SR-A^−/−^ NOD mice were stimulated with poly(I∶C) (0, 10, 50 µg/ml, 0.5 ml). After 10 h, the concentration of TNF-α (A) and after 5 h, the concentration of IFN-β (B) in the culture supernatant were measured by ELISA. (n = 7, mean ± SD).

To determine whether poly(I∶C)-induced acceleration of T1D was associated with immune deviation, we analyzed in vitro production of type I IFNs from BMDCs in the presence of poly(I∶C). Tumor necrosis factor (TNF)-α production was significantly increased in SR-A^−/−^ NOD BMDCs 10 h after stimulation by poly(I∶C) 50 µg/ml, compared with no stimulation by poly(I∶C) (* *p* value = 0.0379 by Mann–Whitney *U* test) (**Figure 8A**). TNF-α production was significantly increased in SR-A^−/−^ NOD BMDCs than in NOD BMDCs after stimulation by poly(I∶C) 10 µg/ml and 50 µg/ml, respectively (* *p* value = 0.0379 by Mann–Whitney *U* test; poly(I∶C) 10 µg/ml, * *p* value = 0.0111 by Mann–Whitney *U* test; poly(I∶C) 50 µg/ml) (**Figure 8A**). IFN-β production showed no dose-dependent increase in NOD BMDCs and SR-A^−/−^ NOD BMDCs at 5 h (**Figure 8B**).

### Pathogenic cells are increased in SR-A^−/−^NOD mice treated with poly(I∶C)

**Figure 9:**
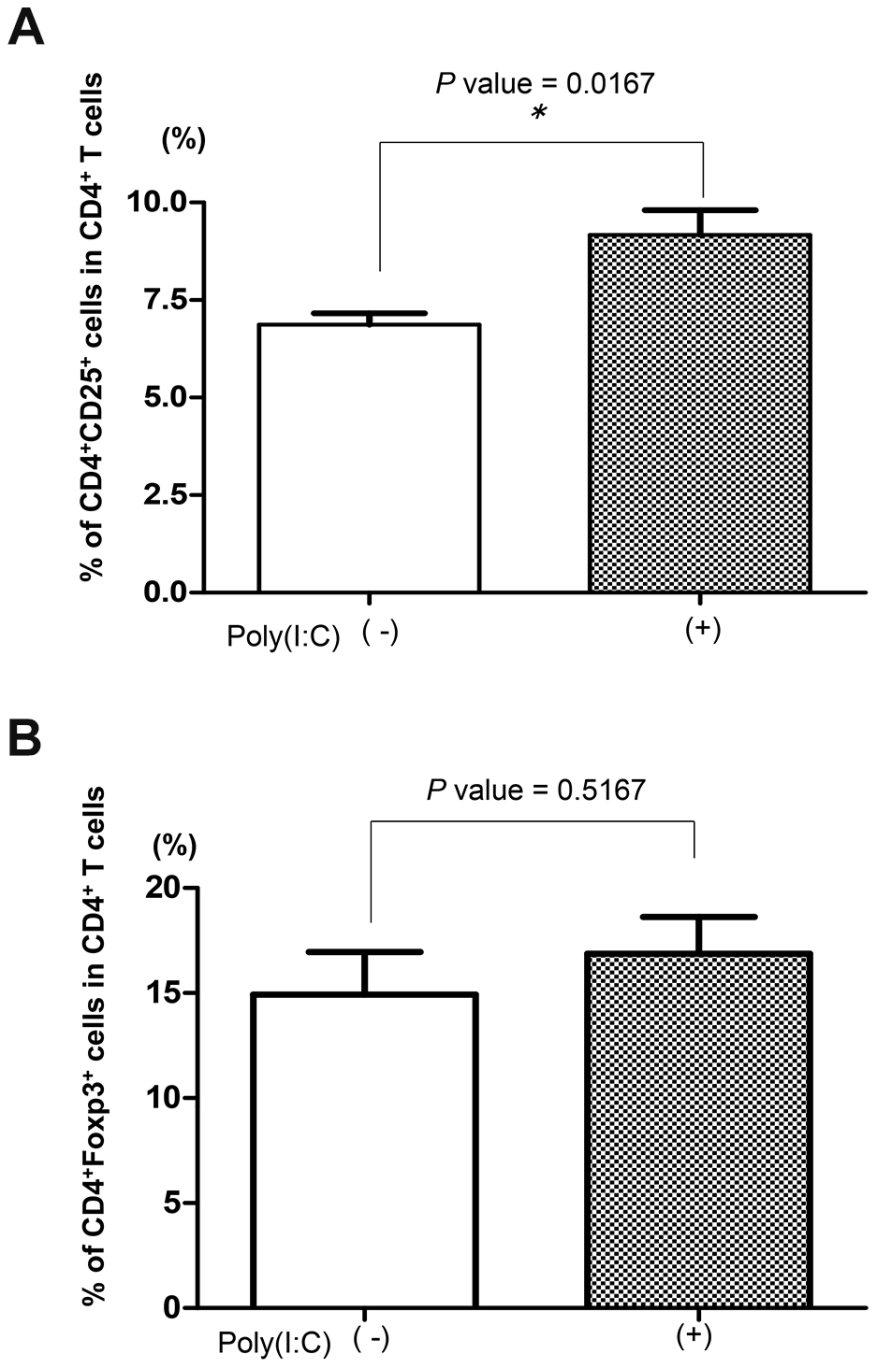
Increased pathogenic T cells in SR-A^−/−^ NOD mice treated with poly(I∶C). (A) The number (percentage) of CD4^+^CD25^+^ T cells was higher in SR-A^−/−^ NOD mice (6 weeks of age) treated with poly(I∶C) (n = 3) than in untreated SR-A^−/−^ NOD mice (n = 7), suggesting that CD4^+^CD25^+^ T cells mainly comprised pathogenic T cells. mean ± SD. * *p* value = 0.0167. (B) The number (percentage) of CD4^+^Foxp3^+^ T cells in SR-A^−/−^ NOD mice (6 weeks of age) treated with poly(I∶C) (n = 3) was similar to that in untreated SR-A^−/−^ NOD mice (n = 7), mean ± SD. *p* value = 0.5167.

The percentage of CD4^+^CD25^+^ pathogenic T cells in splenocytes of SR-A^−/−^ NOD mice (6 weeks old) treated with poly(I∶C) were significantly increased compared with those in untreated SR-A^−/−^ NOD mice (* *p* value = 0.0167 by Mann–Whitney *U* test) (**Figure 9A**). In contrast, the number of CD4^+^Foxp3^+^ regulatory T cells in splenocytes from SR-A^−/−^ NOD mice (6 weeks old) treated with poly(I∶C) was similar to that in untreated SR-A^−/−^ NOD mice (**Figure 9B**). These results suggested a possible mechanism for the acceleration of diabetes development induced in SR-A^−/−^ NOD mice by poly(I∶C) treatment.

## Discussion

Our studies demonstrated that SR-A^−/−^ NOD mice showed significant suppression of diabetes development compared with NOD mice. We also found that diabetes progression in SR-A^−/−^ NOD mice treated with poly(I∶C) (100 µg) was significantly accelerated compared with that in NOD mice treated with poly(I∶C) (100 µg), which in fact showed significant suppression of diabetes development compared with untreated NOD mice.

Recently, it was reported that SR-A is a cell surface receptor for dsRNA and that extracellular dsRNA is recognized and internalized by SR-A. It was reported that diabetes development was completely prevented in MyD88^−/−^ NOD mice, whereas the deletion of TLR3 could not suppress diabetes development in NOD mice [15], [16]. To investigate whether SR-A plays a crucial role in TLR3 recognition of dsRNA, we studied diabetes progression in SR-A^−/−^ NOD or wild type NOD mice in the presence or absence of poly(I∶C) treatment.

To determine the mechanism of suppression of diabetes development in SR-A^−/−^ NOD mice, we investigated the cytokine production by, and phenotype of, splenocytes. Higher production of IFN-γ was detected in splenocytes from SR-A^−/−^ NOD mice than in those from NOD mice (**Figure 6B**). It has been reported that immunization with complete Freund's adjuvant and Bacillus Calmette–Guerin vaccine can prevent T1D in NOD mice, suggesting that IFN-γ might play a critical role in the protection [20], [21]. Previous reports showed that IFN-γ has protective effects in models of autoimmune disease including in experimental autoimmune encephalomyelitis (EAE) model mice, although EAE is regarded as a T helper (Th)-17 (IL-17) and Th-1 (IFN-γ)-dependent autoimmune disease [22]–[25]. Recent studies in NOD mice suggested that IL-17-producing Th-17 cells also play a crucial role in the pathogenesis of T1D [26]–[29]. However, diabetes-resistant SR-A^−/−^ NOD mice showed no impairment of IL-17 production compared with NOD mice (**Figure 6C**). In addition, neither CD4^+^CD25^+^ pathogenic T cells nor CD4^+^Foxp3^+^ Treg cells were increased in SR-A^−/−^ NOD mice (**Figure 6A**). Overall, our results suggested that IFN-γ might have protective effects in SR-A^−/−^ NOD mice.

Next, we investigated the influence of the deletion of SR-A on diabetes progression in SR-A^−/−^ NOD mice treated with poly(I∶C). We expected that diabetes progression in SR-A^−/−^ NOD mice treated with poly(I∶C) (100 µg) would be further suppressed. Unexpectedly, diabetes progression was accelerated compared with that in NOD mice treated with poly(I∶C) (100 µg), which showed significant suppression of diabetes development compared with untreated NOD mice (**Figure 7B**), and was almost the same as that in untreated NOD mice. Unlike NOD mice, low-dose poly(I∶C) led to acceleration, not suppression, of diabetes progression in SR-A^−/−^ NOD mice.

Previous reports showed that injection of the dsRNA mimic poly(I∶C) (200 µg) into Treg-deficient CD28^−/−^ NOD mice at 8 weeks of age led to rapid development of diabetes within 1–6 days after administration that showed a fulminant T1D-like phenotype [30], [31]. To strongly mimic an acute RNA virus infection such as an enterovirus infection and clearly make sure the effect on immunity, we also used high-dose (300 µg) poly(I∶C) dsRNA analogue treatment of both SR-A^−/−^ NOD mice and NOD mice, which showed no decrease in Tregs. Injection of high-dose poly(I∶C) into NOD mice led to acceleration of diabetes progression similar to that in untreated NOD mice. Interestingly, in our study, SR-A^−/−^ NOD mice treated with high-dose poly(I∶C) showed significantly accelerated diabetes development at a younger age compared with untreated diabetes-resistant SR-A^−/−^ NOD mice (**Figure 7A**).

Pathogenic CD4^+^CD25^+^ activated T cells were significantly increased in SR-A^−/−^ NOD mice treated with poly(I∶C) compared with those without poly(I∶C) treatment (**Figure 9**). It has been reported that low-dose (100 µg), but not high-dose (300 µg) poly(I∶C) injection to wild type NOD mice leads to an increase in both pathogenic T cells and Tregs, resulting in a fine balance of both populations [32]. Therefore, we predicted that injection of high-dose poly(I∶C) to NOD mice might induce a shift of the balance toward pathogenic T cells, resulting in more aggressive diabetes compared with NOD mice treated with low-dose poly(I∶C). However, in the SR-A^−/−^ NOD mice, even low-dose poly(I∶C) treatment led to a shift of the balance towards pathogenic T cells.

A previous study using reverse transcription–polymerase chain reaction (RT–PCR) showed that there was a time- and concentration-dependent induction of IFN-β and TNF-α when cells were treated with Ino-RNA [33]. As shown in **Figure 8A**, our study demonstrated that the in vitro cytokine production from BMDCs in response to poly(I∶C) showed a significantly dose-dependent increase in TNF-α production at 10 h in SR-A^−/−^ NOD but not NOD mice. The increase of TNF-α production in SR-A–deficient mice suggests that SR-A signaling negatively affects TNF-α production through the TLR3 pathway after poly(I∶C) stimulation. Our result is consistent with previous reports that SR-A is a potential TNF-α suppressor [34]–[37]. In contrast, as shown in **Figure 8B**, IFN-β production showed no dose-dependent increase in either SR-A^−/−^ NOD or NOD mice, suggesting that IFN-β production is independent of SR-A.

Several lines of evidence support a role for SR-A in peripheral tolerance [9], [38]–[42]. For example, it has been reported that SR-A deficiency is protective against diabetic nephropathy in NOD mice [38], against autoantibody-dependent arthritis and EAE [39], [40]. Previous reports showed that TNF-α administration in older mice prevented autoimmune diabetes in NOD mice and that TNF-α expression from a young age in TNF-α transgenic NOD mice accelerated diabetes progression [43]–[45]. In our study, TNF-α production by BMDC from young SR-A^−/−^ NOD mice showed a marked dose-dependent increase in the presence of poly(I∶C). Our results suggested that this increase might participate in the significant acceleration of T1D development caused by high-dose poly(I∶C) treatment in young SR-A^−/−^ NOD mice (**Figure 7A**). These results suggest that poly(I∶C) binds to surface-expressed SR-A and communicates a negative signal to endosomal TLR3.

**Figure 10:**
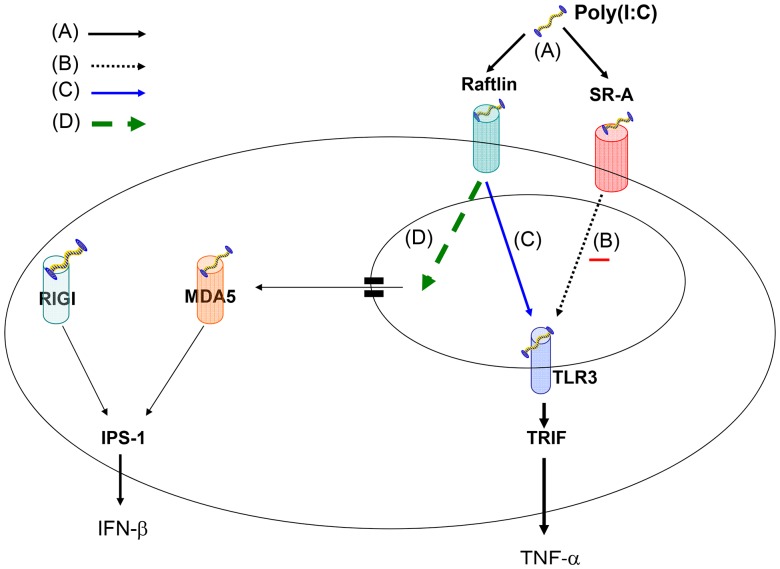
Hypothesized TLR3 RNA-sensing system. (A) Extracellular dsRNA binds SR-A or raftlin on cell surface and enters via endocytosis. (B) SR-A communicates a negative signal to endosomal TLR3, which binds dsRNA and induces TNF-α production via TRIF. (C) Raftlin communicates a positive signal to endosomal TLR3, which binds dsRNA and induces TNF-α production via TRIF. (D) dsRNA escapes the endosome and is detected in the cytoplasm by MDA5 and/or RIG-I, which induces IFN-β production via IPS-1 without TLR3 signaling.

Interestingly, a more recent study showed that raftlin is another cell surface receptor for RNA and that raftlin-mediated endocytosis is important for the TLR3 RNA-sensing system [46]. A previous study reported that EAE was ameliorated and IL-17 production reduced in raftlin^−/−^ mice [47]. Taking these findings together with our study, we hypothesize that there are three pathways for dsRNA signaling as shown in **Figure 10**. Extracellular dsRNA binds SR-A or raftlin on the cell surface and enters via endocytosis. SR-A communicates a negative signal to endosomal TLR3 and raftlin communicates a positive signal to endosomal TLR3, which induces TNF-α production via TRIF [11]. dsRNA escapes the endosome and is detected in the cytoplasm by MDA5 and/or RIG-I, which induces IFN-β production via IPS-1 without signaling TLR3 [48]. We speculate that if a negative signal cannot be conveyed from SR-A to TLR3, TNF-α production might be increased through the raftlin-mediated TLR3 signaling pathway, and that this might be involved in the acceleration of T1D development in young SR-A^−/−^ NOD mice treated with poly(I∶C). In an infection, we consider that the balance between the SR-A signal and the raftlin signal might play a crucial role in diabetes progression and that strong stimulation might lead to an increase in the raftlin signal, resulting in the acceleration of T1D development. Thus, SR-A may play an unfavorable role in steady-state conditions and a protective role in a mild infection.

In conclusion, our studies demonstrate that diabetes progression in untreated SR-A^−/−^ NOD mice was significantly suppressed compared with that in untreated NOD mice. However, low-dose poly(I∶C) treatment accelerated diabetes progression in SR-A^−/−^ NOD mice and suppressed it in NOD mice. Our findings suggest that SR-A on APCs such as DCs might have dual effects on T1D progression and be an important target for improving therapeutic strategies for T1D.

## Supporting Information

Figure S1 **Cyclophosphamide-induced diabetes.** Cyclophosphamide (CY) (200 mg/kg body weight) was injected intraperitoneally into 8-week-old male NOD mice (n = 5) and SR-A^−/−^ NOD mice (n = 5) twice, with a 2-week interval between injections. All mice were nondiabetic before injection. Administration of CY accelerated diabetes onset and 80% (4/5) of NOD mice developed overt diabetes within 50 days of the initial injection. In contrast, 20% (1/5) SR-A^−/−^ NOD mice showed diabetes. Diabetes incidence was markedly lower in SR-A^−/−^ NOD mice than in NOD mice in CY-induced diabetes model. CY-induced diabetes model showed similar results to the spontaneous SR-A^−/−^ NOD mouse.
(TIF)

Figure S2 **Multiple low-dose STZ (MLDS)–induced diabetes.** STZ (40 mg/kg body weight) was injected intraperitoneally into 8-week-old male NOD mice (n = 5) and SR-A^−/−^ NOD mice (n = 5) daily for five consecutive days for induction of autoimmune diabetes. Multiple low-dose STZ (MLDS)-treated mice were observed for diabetes development for 30 days after initial STZ injection. 40% (2/5) of NOD mice given MLDS administration showed overt diabetes. In contrast, 60% (3/5) of SR-A^−/−^ mice given MLDS administration showed overt diabetes. Diabetes incidence in SR-A^−/−^ NOD mice was almost the same as that in NOD mice in MLDS-induced diabetes model. MLDS-induced diabetes model showed different results to the spontaneous SR-A^−/−^ NOD mouse.
(TIF)

Figure S3 **Serum C-peptide measurement.** Insulin secretion was assessed by serum measurements of causal C-peptide levels in NOD mice (n = 4) or SR-A^−/−^ NOD mice (n = 4) at 7 weeks and 25 weeks of age, respectively. C-peptide was measured using ELISA kit following the protocols provided by the manufacturer (Shibayagi Co Ltd, Shibukawa, Japan). C-peptide levels were significantly higher in 7- and 25-week-old SR-A^−/−^ NOD mice than in NOD mice, respectively (* *p*<0.05 by Mann–Whitney *U* test).
(TIF)

Figure S4 **Histology in NOD mice and SR-A^−/−^ NOD mice treated with poly (I∶C).** The pancreas was harvested from 14-week-old NOD mice and SR-A^−/−^ NOD mice treated with high-dose poly (I∶C), fixed in 10% formalin, embedded in paraffin. Five-micrometer-thick sections were cut, stained with hematoxylin and eosin, and examined by light microscope. Cellular infiltration of mononuclear cells in islets were obviously confirmed in both NOD mice and SR-A^−/−^ NOD mice treated with high-dose poly (I∶C) by histological examination.
(TIF)
