# Supplementary figures and images for: The Dual Role of Scavenger Receptor Class A in Development of Diabetes in Autoimmune NOD Mice
Source: PLoS One. 2014 Oct 24;9(10):e109531. doi: 10.1371/journal.pone.0109531 (PMC4208757; doi:10.1371/journal.pone.0109531)

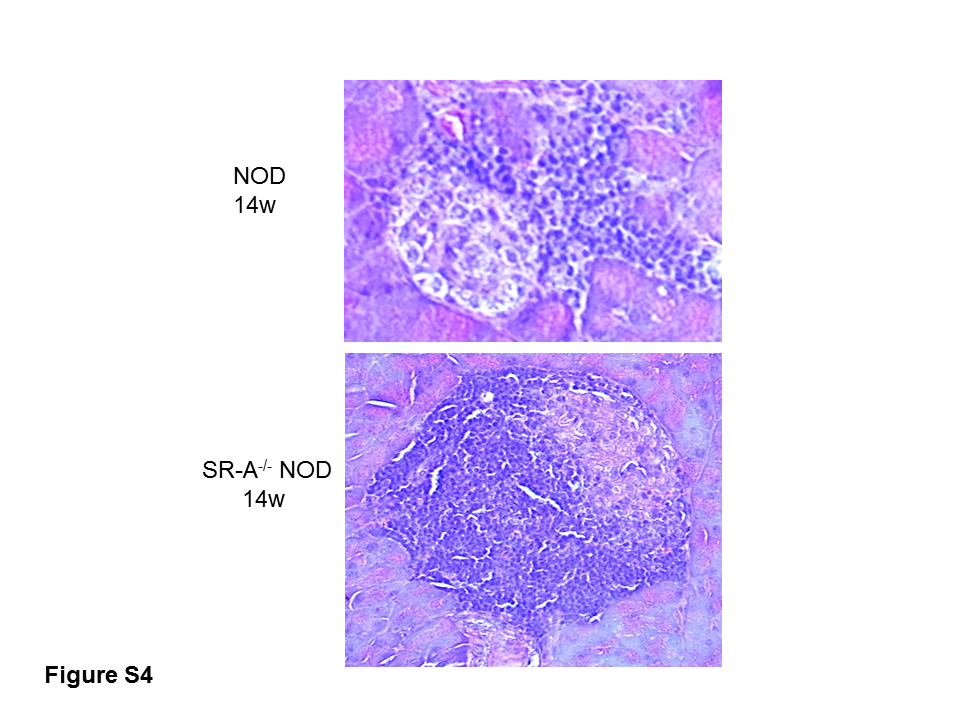

Supplement: Figure S4 — Histology in NOD mice and SR-A−/− NOD mice treated with poly (I∶C). The pancreas was harvested from 14-week-old NOD mice and SR-A−/− NOD mice treated with high-dose poly (I∶C), fixed in 10% formalin, embedded in paraffin. Five-micrometer-thick sections were cut, stained with hematoxylin and eosin, and examined by light microscope. Cellular infiltration of mononuclear cells in islets were obviously confirmed in both NOD mice and SR-A−/− NOD mice treated with high-dose poly (I∶C) by histological examination. (TIF) [file pone.0109531.s004.tif]
